# A stable DNA-free screening system for CRISPR/RNPs-mediated gene editing in hot and sweet cultivars of *Capsicum annuum*

**DOI:** 10.1186/s12870-020-02665-0

**Published:** 2020-10-01

**Authors:** Hyeran Kim, Jisun Choi, Kang-Hee Won

**Affiliations:** Department of Biological Sciences, Kangwon National University, Kangwondaehak-gil 1, Chuncheon, 24341 South Korea

**Keywords:** Pepper genome editing, *Capsicum annuum* CM334, *C. annuum* Dempsey, CRISPR/Cas9, CRISPR/LbCpf1, Pepper leaf protoplasts, Pepper callus protoplasts

## Abstract

**Background:**

DNA-free, clustered regularly interspaced short palindromic repeats (CRISPR)-associated protein (Cas) ribonucleoprotein (RNP)-based genome editing is a simple, convincing, and promising tool for precision crop breeding. The efficacy of designed CRISPR-based genome editing tools is a critical prerequisite for successful precision gene editing in crops.

**Results:**

This study demonstrates that soil-grown leaf- or callus-derived pepper protoplasts are a useful system for screening of efficient guide RNAs for CRISPR/Cas9 or CRISPR/Cas12a (Cpf1). CRISPR/Cas9 or Cpf1 were delivered as CRISPR/RNP complexes of purified endonucleases mixed with the designed single guide RNA, which can edit the target gene, *CaMLO2* in two pepper cultivars with whole genome sequenced, *Capsicum annuum* ‘CM334’ and *C. annuum* ‘Dempsey’. The designed guide RNAs (sgRNAs for Cas9 or crRNAs for Cpf1) are conserved for *CaMLO2* in both CM334 and Dempsey and cleave *CaMLO2* in vitro. CRISPR/Cas9- or /Cpf1-RNP complexes were transfected into purely isolated protoplasts of the hot pepper CM334 and sweet pepper Dempsey by PEG-mediated delivery. Targeted deep sequencing analysis indicated that the targeted *CaMLO2* gene was differentially edited in both cultivars, depending on the applied CRISPR/RNPs.

**Conclusions:**

Pepper protoplast-based CRISPR guide-RNA selection is a robust method to check the efficacy of designed CRISPR tools and is a prerequisite for regenerating edited plants, which is a critical time-limiting procedure. The rapid and convincing selection of guide RNA against a target genome reduces the laborious efforts for tissue culture and facilitates effective gene editing for pepper improvement.

## Background

Clustered regularly interspaced short palindromic repeats (CRISPR)-CRISPR-associated protein (Cas), CRISPR/Cas9 has emerged as the first RNA-guided genome-editing tool to introduce a target mutation in any sequenced genome after being reported as a programmable molecular scissor in 2012 [1]. Since obtaining SpCas9-based CRISPR tools from *Streptococcus pyogenes*, various tools have been developed from different strains, such as *Staphylococcus aureus* Cas9 [2], *Francisella novicida* Cas9 [3], *Streptococcus thermophilus* Cas9 [4], and *Campylobacter jejuni* Cas9 [5]. These developed CRISPR-based tools have been promptly applied to all kinds of research areas from generating knock-out cell lines and organisms to biotechnology of animals [6, 7], plants [8–11], and humans [12–14]_._

CRISPR/Cas12a (Cpf1) has been harnessed for another useful RNA-guided genome editing tool, comprising a single crRNA and a Cpf1 protein that functions in crRNA processing, target-site recognition, and DNA cleavage [15]. Multiple Cpf1 proteins were obtained from various strains, including FnCpf1 from *Francisella tularensis* subsp. *novicida* U112 [16], LbCpf1 from *Lachnospiraceae bacterium* ND 2006, and AsCpf1 from *Acidaminococcus* sp. BV3L6 [15]. Both LbCpf1 and AsCpf1 tools showed successful editing activity in human cells by expression plasmids [15] or Cpf1-ribonucleoprotein (RNP) [17]. The editing effect of both LbCpf1 and AsCpf1 was also successfully validated in soybean and tobacco via Cpf1-RNP [18]. However, plant-specific properties lowered the editing efficiency of mature crRNA-harboring plasmids in rice [19] as well as in soybean and tobacco [18].

To successfully improve target gene editing without any off-target mutation, high-fidelity versions of the Cas9 protein were devised using protein engineering [20, 21]. A guide-RNA format was designed using truncated guide RNA for Cas9 [22], chemically synthesized guide RNA for Cpf1 protein [18, 22], or detoxifying format as a 5′-hydroxyl guide RNA for both Cas9 and Cpf1 [23]. Various delivery methods for CRISPR tools to target organisms are available, including mechanical force-, chemical-, and biological system-based methods. In plants, bombardment [11], Polyethylene glycol (PEG)-mediated [24], and *Agrobacterium*-mediated [25] applications are used to deliver the designed CRISPR tools.

*Capsicum* (pepper) from the family Solanaceae is known to be recalcitrant to genetic manipulation, such as transformation of a target gene and generation of mutant plants. An efficient method for reverse genetic studies in this genus is still lacking although particle bombardment or *Agrobacterium*-mediated transformation has been extensively tested for more than 30 years and similar methods have been developed for other genera of the Solanaceae such as *Solanum* including tomato [26, 27] and *Nicotiana* (tobacco) [28, 29]. Moreover, CRISPR-based genome editing tools have not been reported in pepper.

*C. annuum* ‘CM334’ and ‘Dempsey’ are excellent resources for studying the traits of hot peppers and sweet peppers, respectively, because of their complete genome information [30, 31]. Unlike protoplast systems from model plants such as *Arabidopsis*, tobacco, and rice, which have been extensively used for cell-based studies, pepper protoplasts are prone to collapse due to their sticky property after protoplast isolation. Recently, we successfully induced and maintained pepper-derived calluses from soil-grown leaves of two peppers, the hot pepper CM334 and the sweet pepper Dempsey [32]. These calluses support stable pepper protoplasts to characterize cell-based, functional genetic studies on hot and sweet peppers.

Powdery mildew is a significant fungal disease for greenhouse- and field-grown crops such as tomato and pepper [33, 34]. Among the *mildew resistance locus O* (*MLO*) genes in plants, *AtMLO2* belongs to clade V along with *AtMLO6* and *AtMLO12*, and it is a well-known susceptibility gene that confers broad-spectrum resistance in the null mutant against plant pathogens, especially against powdery mildew [35, 36]. The sequenced genome of *C. annuum* ‘CM334’ presumably contains 18 members of *CaMLO* (Table 1). Previously, *CaMLO2*, an ortholog of *AtMLO2*, was reported as a susceptible gene in peppers against biotrophic and hemibiotrophic pathogens [37, 38]. Due to a lack of targeted mutagenesis in peppers, there is no available pepper *CaMLO* knockout mutant, except for the natural variants.

**Table 1:** MLO proteins in *Arabidopsis thaliana* and *Capsicum annuum*

Species	Gene name	Accession No.	Gene ID	ID source
*Arabidopsis thaliana*	*AtMLO2*	Q9SXB6	AT1G11310	UniProt
	*AtMLO6*	Q94KB7	AT1G61560	
	*AtMLO12*	O80961	AT2G39200	
*Capsicum annuum*	*CA00g74950*	CA00g74950	CA00g74950	Sol Genomics
	*CA02g02090*	CA02g02091	CA02g02092	
	*CA02g04140*	CA02g04141	CA02g04142	
	*CA02g20140*	CA02g20141	CA02g20142	
	*CA02g21400*	CA02g21401	CA02g21402	
	*CA06g10510*	CA06g10511	CA06g10512	
	*CA06g10520*	CA06g10521	CA06g10522	
	*CA06g26150*	CA06g26151	CA06g26152	
	*CA07g17840*	CA07g17841	CA07g17842	
	*CA08g01760*	CA08g01761	CA08g01762	
	*CA08g01780*	CA08g01781	CA08g01782	
	*CA08g05700*	CA08g05701	CA08g05702	
	*CA08g13470*	CA08g13471	CA08g13472	
	*CA09g10750*	CA09g10751	CA09g10752	
	*CA09g10760*	CA09g10761	CA09g10762	
	*CA10g07880*	CA10g07881	CA10g07882	
	*CA11g19200*	CA11g19201	CA11g19202	
	*CA12g10780*	CA12g10781	CA12g10782	

Eighteen of CaMLO proteins in *Capsicum annuum* were obtained by the BLAST tool in Sol Genomics Network (https://solgenomics.net/) based on three AtMLO proteins (AtMLO2, AtMLO6, and AtMLO12) as a query protein sequence. BLAST is performed with the default setting in the database (*Capsicum annuum* cv CM334 Genome protein sequences (release 1.55). Accession No. and Gene ID were retrieved from sequence resource sites (*Arabidopsis thaliana*, https://www.uniprot.org/;*Capsicum annuum*, https://solgenomics.net/)

Here, we present a DNA-free, genome-editing method in two pepper cultivars, *C. annuum* ‘CM334’ and ‘Dempsey’, using preassembled SpCas9 or LbCpf1 with a single guide RNA RNP, CRISPR/Cas9-RNP or CRISPR/LbCpf1-RNP, respectively. To test whether CRISPR-RNP tools can be screened in cellular systems of two peppers, we delivered CRISPR/Cas9-RNPs or LbCpf1-RNPs to pepper protoplasts isolated from soil grown Dempsey leaf and proliferative CM334 callus and analysed insertion and deletion (indel) frequencies and patterns at the target *CaMLO2* gene. Pepper protoplast-based guide RNA screening is thus a starting point to evaluate the efficacy of designed CRISPR systems for further investigation of a gene of interest in the generation of stable transgenic peppers.

## Results

### PEG-mediated CRISPR-RNP delivery in pepper protoplasts

**Fig. 1:**
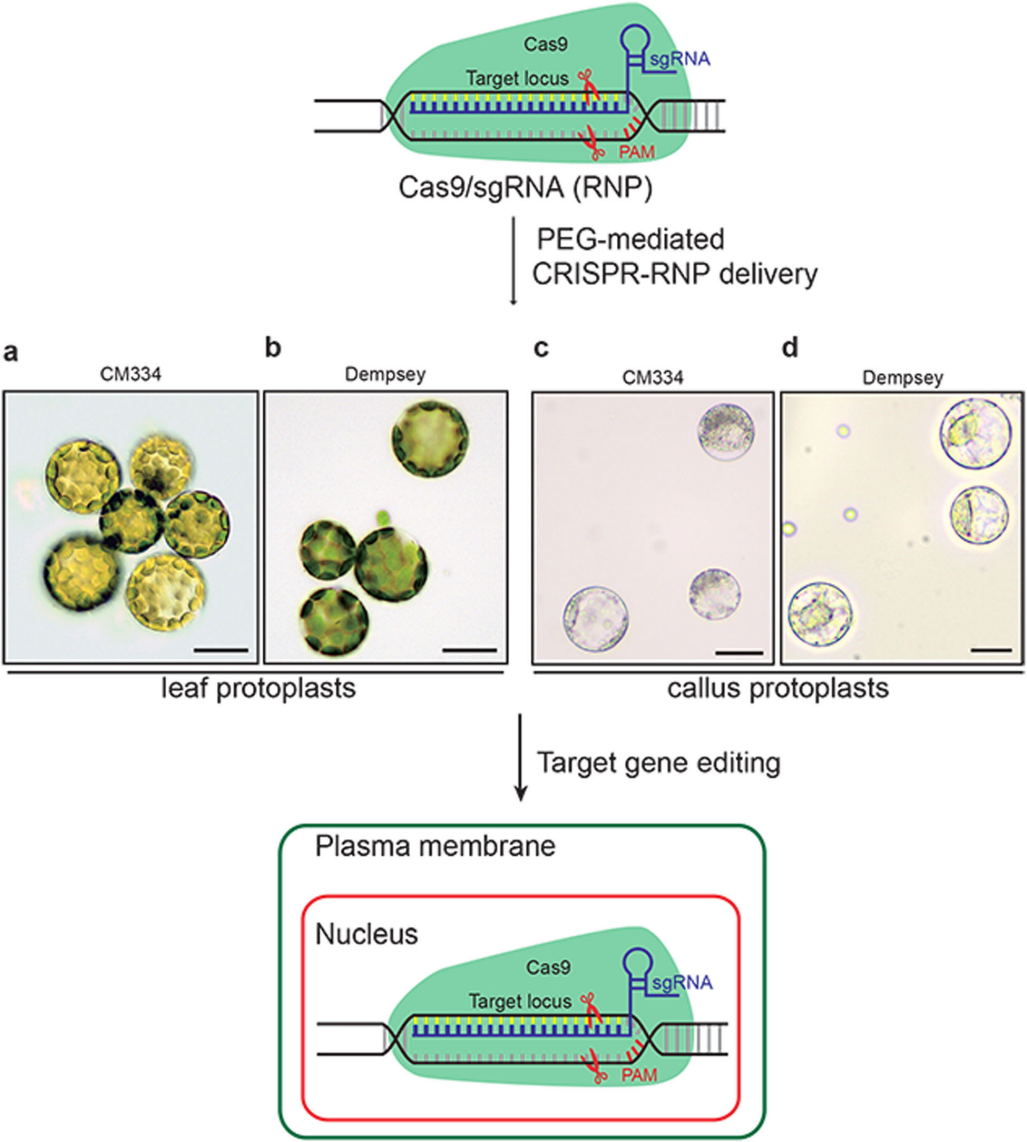
Schematic overview of clustered regularly interspaced short palindromic repeats (CRISPR)-mediated genome editing in pepper protoplasts. Preassembled CRISPR-RNP complexes (for example, CRISPR/Cas9 RNP) were delivered to purely isolated protoplasts from the leaf or callus of CM334 and Dempsey peppers by PEG-mediated transfection. The delivered CRISPRs RNP was targeted to the nucleus of the pepper protoplasts and subsequently cleaved the target locus. Scale bars, 20 μm

To assess whether CRISPR-RNPs can be delivered to protoplasts of CM334 and Dempsey, we isolated protoplasts from the two pepper cultivars grown in soil (Fig. 1a and b). Dempsey leaf protoplasts were stable enough to be applied in CRISPR-RNPs. In contrast, the CM334 leaf protoplast isolates, although pure (Fig. 1b), were unstable and challenging to harvest after delivery of CRISPR/Cas9-RNP. Previously, we established leaf-derived calluses from soil-grown CM334 and Dempsey, which can provide stable protoplasts for cell-based studies [32]. We thus explored callus-derived pepper protoplasts as a platform for screening an appropriate gene editing tool (Fig. 1c and d).

### In vitro validation of the designed CRISPR RNPs for Cas9 and LbCpf1

**Fig. 2:**
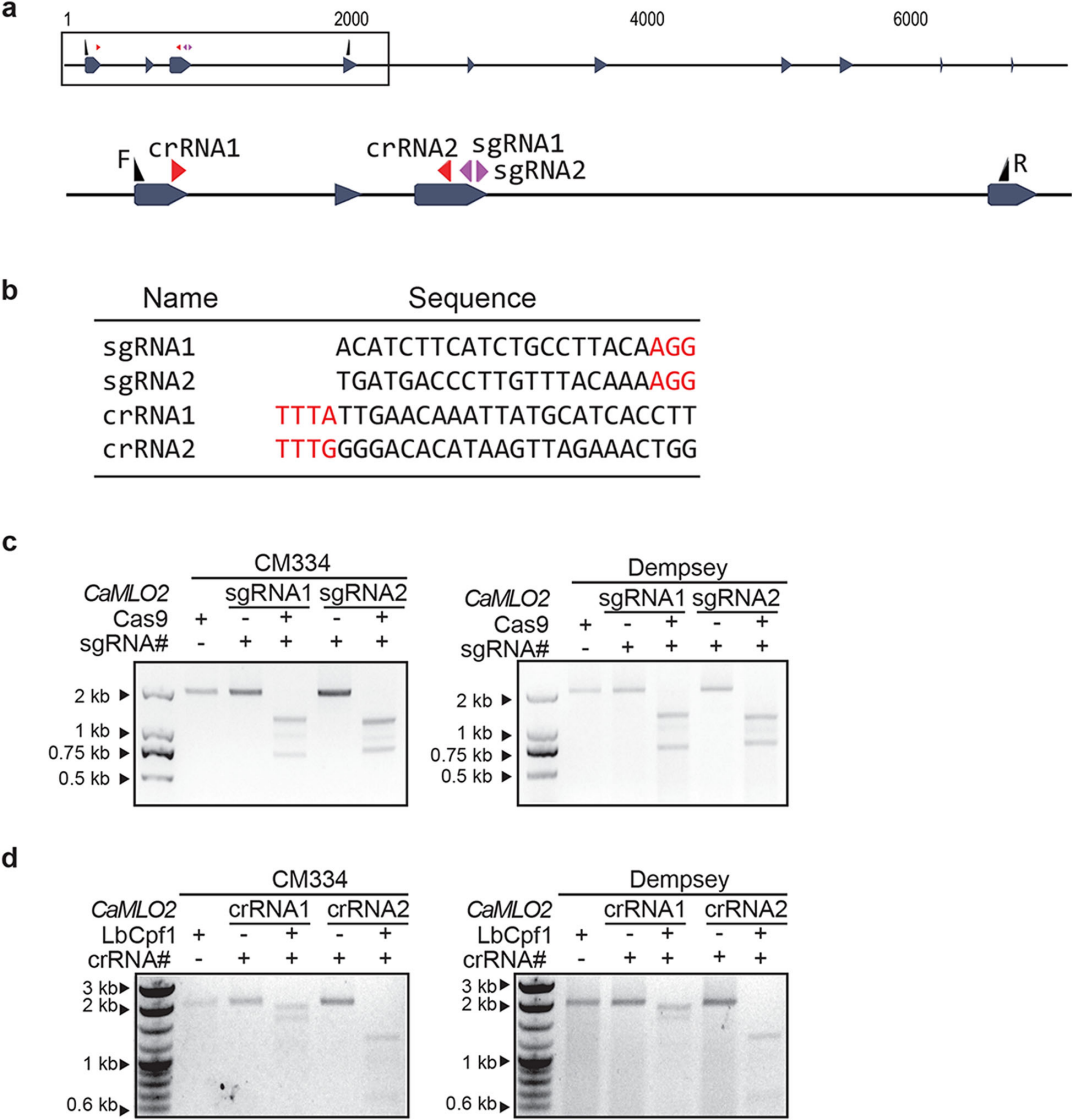
In vitro cleavage assay for CRISPR/Cas9 or CRISPR/Cpf1 RNP-mediated *CaMLO2* gene in two peppers. **a** Target locus of *CaMLO2* gene, four designed guide RNAs (sgRNA1 and sgRNA2 for Cas9 and crRNA1 and crRNA2 for LbCpf1) and a specific primer pair, F and R. **b** Target sequences of the four guide RNAs. **c** In vitro cleavage assay with preassembled Cas9-only as a control, Cas9-sgRNA1 and Cas9-sgRNA2 for *CaMLO2* gene of CM334 or Dempsey. **d** In vitro cleavage assay with preassembled LbCpf1-only as a control, LbCpf1-crRNA1 and LbCpf1-crRNA2 for *CaMLO2* gene of CM334 and Dempsey

The genomic region of the *CaMLO2* gene in both CM334 and Dempsey was firstly analyzed by Sanger sequencing to confirm the conserved exon sequences. We subsequently designed two sgRNAs on the 3rd exon for Cas9 proteins (sgRNA1: 5′-ACATCTTCATCTGCCTTACA-3′ and sgRNA2: 5′ TGATGACCCTTGTTTACAAA-3′) and two crRNAs on the 1st and 3rd exon for LbCpf1 (crRNA1: 5′-TTGAACAAATTATGCATCACCTT-3′ and crRNA2: 5′-GGGACACATAAGTTAGAAACTGG-3′) (Fig. 2a and b). We selected specific guide RNAs without up to two nucleotide mismatches based on the entire homology search against the current pepper reference genome using Cas-Designer from RGEN tools [39]. We performed in vitro cleavage assays to validate the activity of CRISPR-RNP complexes of Cas9-sgRNA and LbCpf1-crRNA, consisting respectively of recombinant Cas9 and LbCpf1 proteins and in vitro transcribed guide RNAs, in two pepper cultivars. The target fragment of *CaMLO2* was amplified with a primer pair, F and R, denoted in Fig. 2a and Table 3. Cas9-RNPs and LbCpf1-RNPs efficiently cleaved the target regions of *CaMLO2* in both CM334 and Dempsey in vitro*,* as expected (Fig. 2c and d).

### Analysis of CRISPR/Cas9-RNP in Dempsey leaf protoplasts

**Fig. 3:**
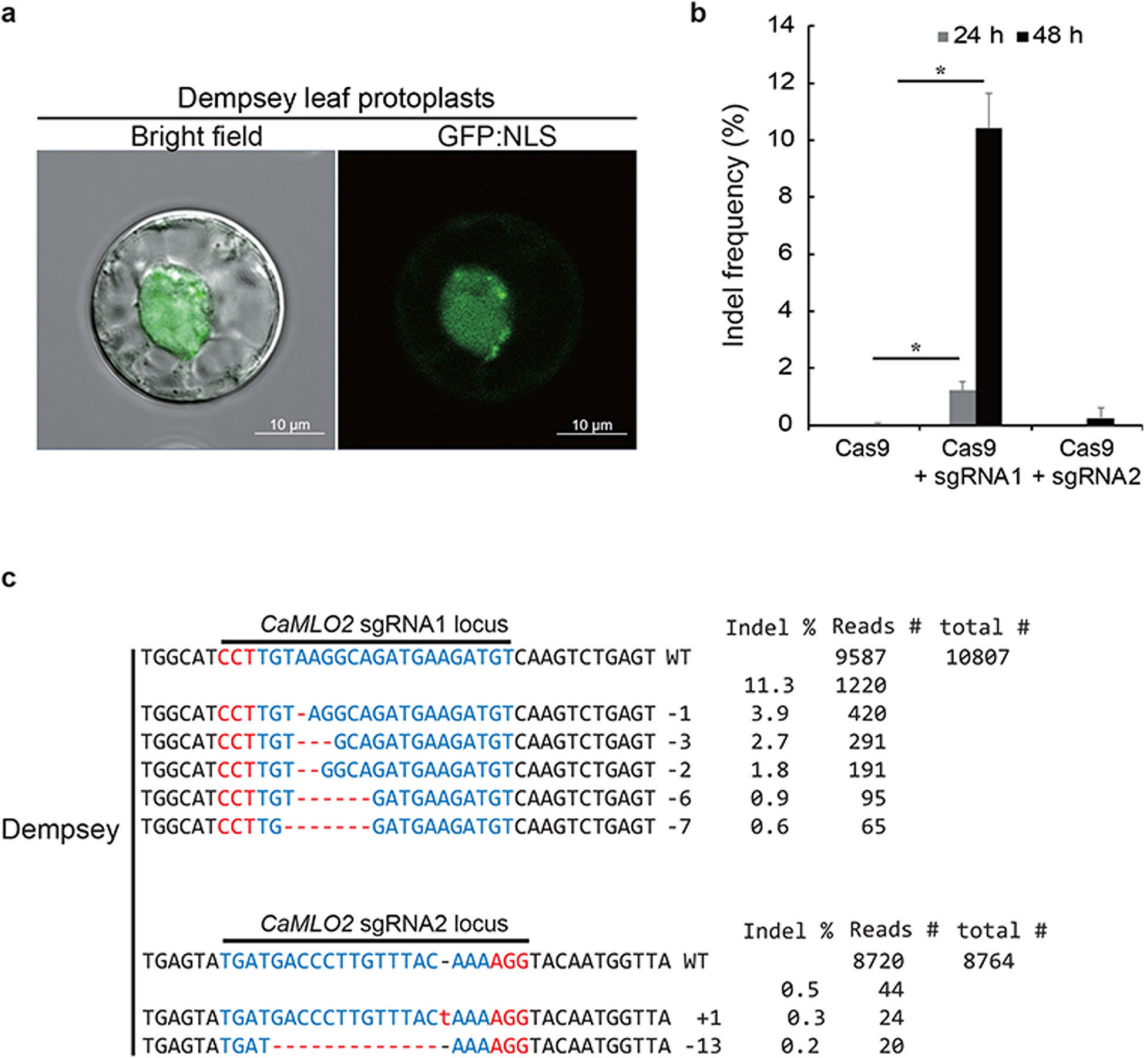
Dempsey leaf protoplasts for CRISPR/Cas9 RNP-mediated *CaMLO2* editing. **a** Dempsey leaf protoplast expressed nucleus-targeted GFP:NLS. **b** Indel frequency of Cas9-sgRNA1 and Cas9-sgRNA2 transfected into Dempsey leaf protoplasts. Vertical bars represent the mean ± standard deviation (*n* = 3). Asterisk indicates significant different at *P* < 0.01 compared with Cas9-only based on ANOVA. **c** Indel patterns of Cas9-sgRNA1 and Cas9-sgRNA2 editing of *CaMLO2* loci. Total reads were obtained by targeted deep sequencing. Indel frequency (%) was calculated as the number of measured reads divided by the number of total reads. Red, PAM sequences; Blue, CRISPR target sequence; Red dash lines and -, deleted nucleotides; Red letters and +, inserted nucleotides

We investigated PEG-mediated CRISPR/Cas9 RNP delivery into protoplasts isolated from CM334 and Dempsey leaves. First, we tested whether pepper protoplasts were transiently transfected with a conventional plasmid harboring a GFP:NLS expressing cassette as a nuclear marker. The Dempsey leaf protoplasts were efficiently transfected and showed GFP signals in the nucleus after 24 h of incubation (Fig. 3a). However, CM334 leaf protoplasts were not stable enough to express the transfected plasmid or CRISPR/Cas9 RNP. Moreover, Dempsey leaf protoplasts were successfully transfected and maintained until the detection of transfected GFP plasmid or subsequent genotype analysis. Dempsey protoplasts transfected with CRISPR/Cas9-RNP were harvested at 24 h and 48 h of incubation (Fig. 3b). These were used to extract the genomic DNA and perform targeted deep sequencing to analyze the indel frequencies and patterns at the target sites in the *CaMLO2* gene. Indels using CRISPR/Cas9-RNP at 24 h were marginally captured at the target sites with frequencies of either 1.23% for sgRNA1 or 0.02% for sgRNA2 in *CaMLO2* at 24 h (Fig. 3b). After 48 h of incubation, indels were dramatically increased and differentially captured at the target sites with 11.3% for sgRNA1 or 0.5% for sgRNA2 (Fig. 3b). Most of the indels induced at the *CaMLO2* gene using Cas9-sgRNA1 complexes were deletions of several nucleotides located 3 bp upstream of PAM (as 5′-CCT-3′), compared with Cas9-sgRNA2 (Fig. 3c). This result indicated that the designed sgRNA1 containing CRISPR/Cas9-RNP complex is more effective to edit the *CaMLO2* gene than sgRNA2. All results demonstrated that Dempsey leaf protoplasts are a stable cellular system that can be used to validate an efficient CRISPR/Cas9-RNP for target gene editing in sweet pepper.

### Analysis of CRISPR/Cas9-RNP in CM334 callus protoplasts

**Fig. 4:**
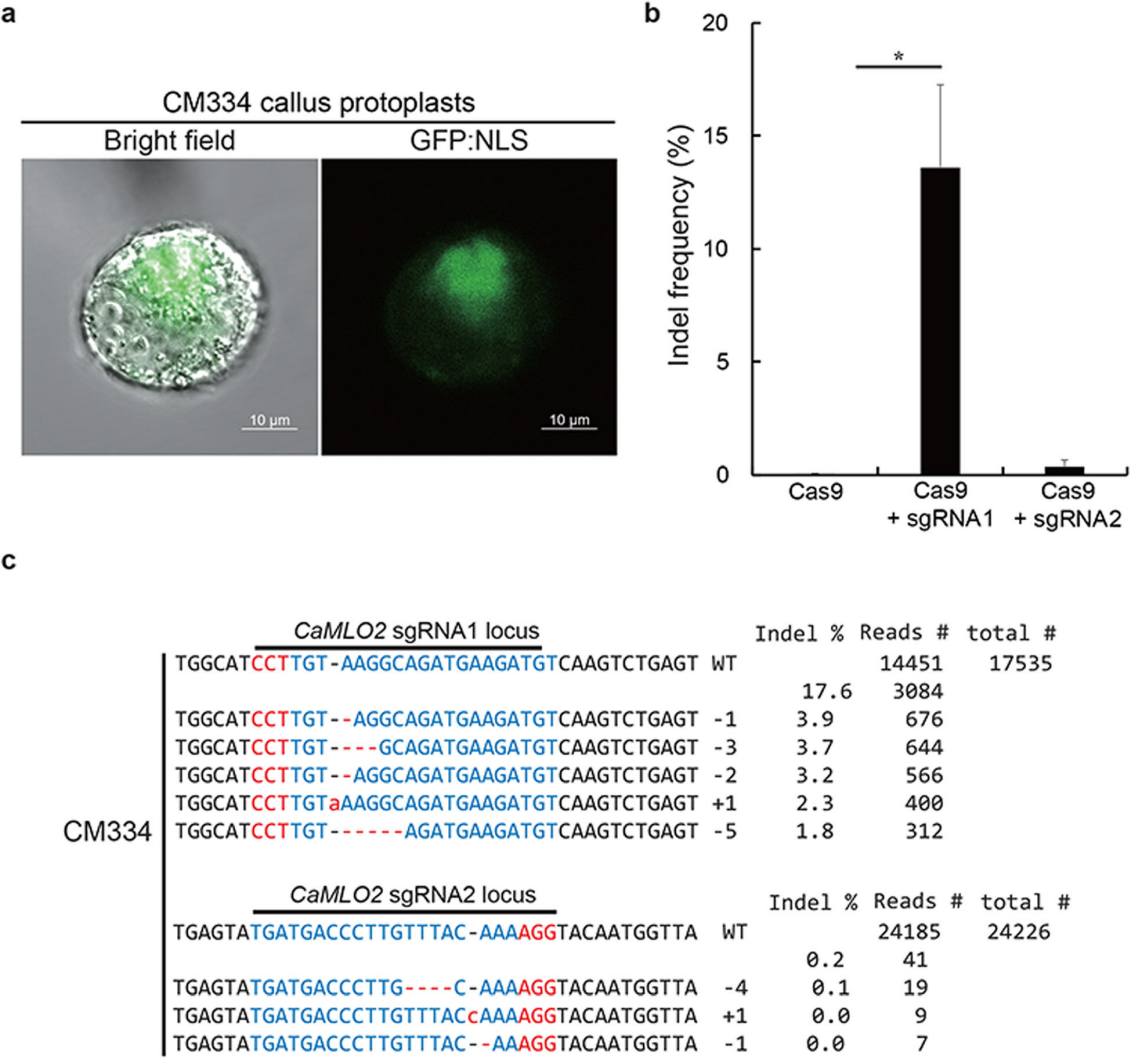
CM334 callus protoplasts for CRISPR/Cas9 RNP-mediated *CaMLO2* editing. **a** CM334 callus protoplast expressed the nucleus-targeted GFP:NLS. **b** Indel frequency of Cas9-sgRNA1 and Cas9-sgRNA2 transfected into CM334 callus protoplasts. Vertical bars represent the mean ± standard deviation (*n* = 4). Asterisk indicates significant difference as *P* < 0.01 compared with Cas9-only based on ANOVA. **c** Indel patterns of Cas9-sgRNA1 and Cas9-sgRNA2 editing of *CaMLO2* loci. Total reads were obtained by targeted deep sequencing. Indel frequency (%) was calculated as the number of measured reads divided by the number of total reads. Red, PAM sequences; Blue, CRISPR target sequence; Red dash lines and -, deleted nucleotides; Red letters and +, inserted nucleotides

To explore a cellular system for CM334, we used the established propagating callus lines from CM334 leaves. Pure isolates of CM334 callus-derived protoplasts were obtained (Fig. 1c) and transfected with a plasmid containing the GFP:NLS expressing cassette. The transfected CM334 callus protoplasts expressed and distinctively demonstrated GFP signals in the nucleus after 48 h of incubation (Fig. 4a). Therefore, we further explored whether CM334 callus protoplasts carry CRISPR/Cas9-RNP and validated the active guide RNAs as a screening system to evaluate CRISPR-RNPs. We delivered the preassembled Cas9 proteins together with the designed sgRNA1 and sgRNA2 as RNP complexes into CM334 callus protoplasts. The transfected CM334 callus protoplasts were incubated for 48 h before analyzing the editing efficacy. The Cas9-only control did not induce any mutation at the target locus of *CaMLO2*, whereas the Cas9-sgRNA1 complexes induced 17.6% of indel mutations at the target site (Fig. 4b and c). However, the activity of Cas9-sgRNA2 complexes (approximately 0.2%) was less efficient in inducing indel mutations, unlike Cas9-sgRNA1 complexes, despite the sparse indel patterns (Fig. 4b and c). The active Cas9-sgRNA1 complexes mainly conferred deletions of several nucleotides. These results indicate that Cas9-sgRNA1 RNP complexes actively edit *CaMLO2* and could be used in further regeneration procedures to produce *CaMLO2* mutations in the hot pepper CM334.

### Analysis of CRISPR/LbCpf1-RNP in CM334 callus protoplasts

**Fig. 5:**
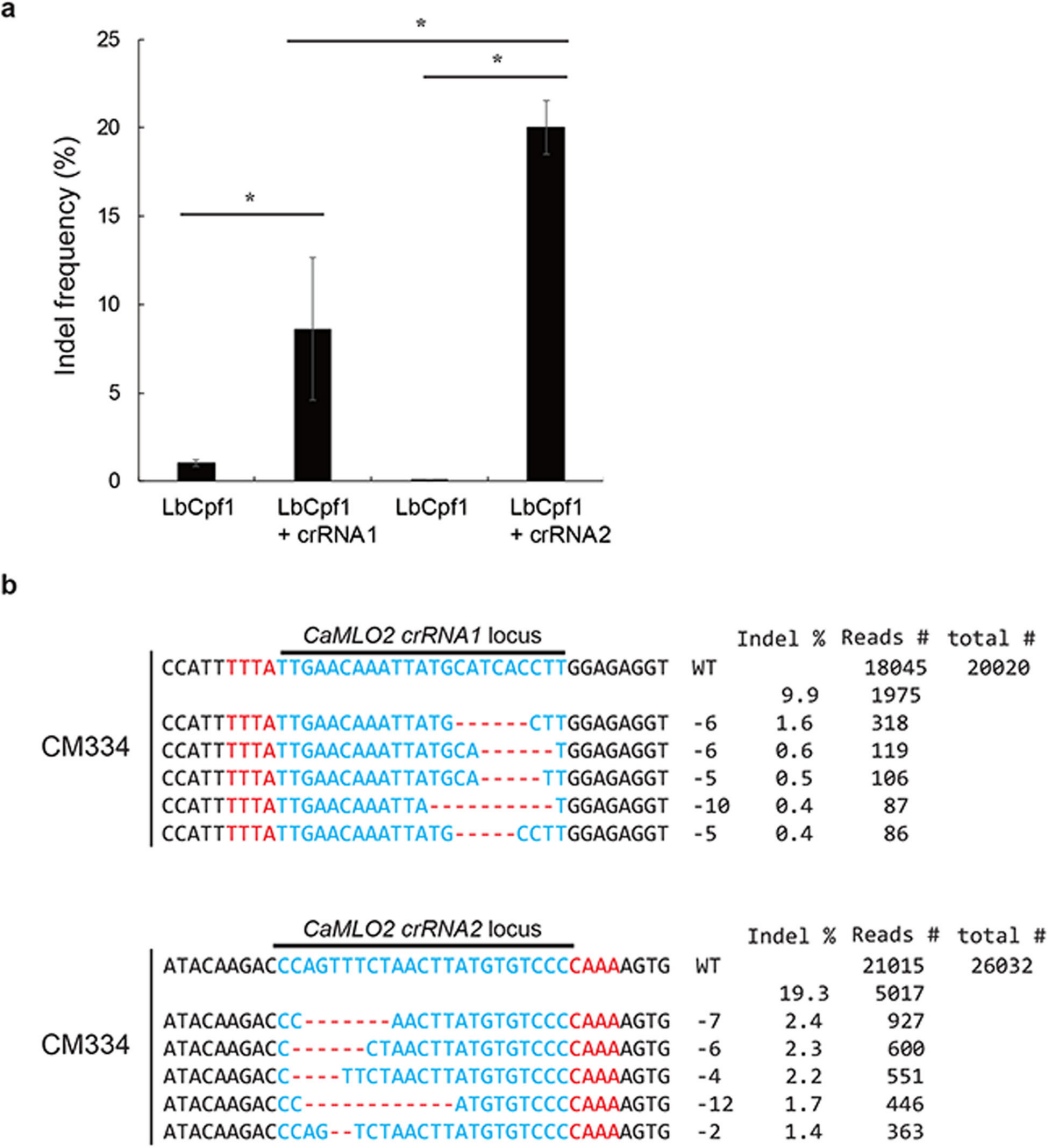
CM334 callus protoplasts for CRISPR/LbCpf1 RNP-mediated *CaMLO2* editing. **a** Indel frequency of LbCpf1-crRNA1 and LbCpf1-crRNA2 transfected into CM334 callus protoplasts at 48 h post-incubation. Vertical bars represent the mean ± standard deviation (*n* = 3). The asterisk indicates significant difference as *P* < 0.01 compared with LbCpf1 only based on ANOVA. **b** Indel patterns of LbCpf1-crRNA1 and LbCpf1-crRNA2 editing of *CaMLO2* loci. Total reads were obtained by targeted deep sequencing. Indel frequency (%) was calculated as the number of measured reads divided by the number of total reads. Red, PAM sequences of TTTN; Blue, CRISPR target sequence; Red dash lines and -, deleted nucleotides

We examined the activity of LbCpf1-RNP in CM334 callus protoplasts via PEG-mediated delivery. The complexes of LbCpf1-crRNA1 and LbCpf1-crRNA2 were successfully delivered into CM334 callus protoplasts. The transfected callus protoplasts were stable enough to evaluate indel mutations, unlike the leaf protoplasts. Protoplasts with LbCpf1 only were used as a control for LbCpf1 RNP transformation. The protoplasts transfected with LbCpf1-crRNA complexes exhibited indel frequencies of 9.9% for LbCpf1-crRNA1 and 19.3% for LbCpf1-crRNA2 (Fig. 5a). The designed LbCpf1-crRNA2 activity was two-fold higher than that of LbCpf1-crRNA1 based on the induced indel frequencies at the *CaMLO2* gene loci. As previously reported with the distinct activities of designed guide RNAs in soybean, cabbage, and petunia [18, 40, 41], the two designed crRNAs of *CaMLO2* exhibited differential editing efficacy based on unknown properties of the sequence context of a target gene.

The indel patterns of targeted LbCpf1-crRNA1 complexes varied at the *CaMLO2* gene locus in CM334 callus protoplasts, with several deletions of nucleotides (− 6, − 5, and − 10) in the first five ranked reads (Fig. 5b). The other targeted LbCpf1-crRNA2 complexes also showed varied indel patterns with distinct deletions of nucleotides (− 7, − 6, − 4, − 12, and − 2) for *CaMLO2* crRNA2 locus in the first five ranked reads (Fig. 5b). The validated LbCpf1-crRNA2 complexes can be used for *CaMLO2* editing in CM334. These data indicate that the established stable pepper protoplasts are robust systems for screening of effective CRISPR tools that can be utilized for precision editing in peppers.

## Discussion

Here, we demonstrated that Dempsey leaf protoplasts and CM334 callus protoplasts are stable and robust cell-based systems for evaluating the CRISPR tools Cas9 and LbCpf1. Using the designed guide RNAs, sgRNAs for Cas9 and crRNAs for LbCpf1, we can examine whether the applied guide RNAs are active enough to induce gene editing specifically at the target sites. In Dempsey, Cas9-sgRNA1 RNP has an indel frequency of 11.3%. The designed Cas9-sgRNA1 complex can thus be used to generate *CaMLO2-*edited Dempsey pepper with broad-spectrum resistance to powdery mildew [38] and hemibiotrophic bacterial pathogens such as *Xanthomonas campestris* pv. *vesicatoria* and *Pseudomonas syringae* pv. *tomato* (*Pst*) DC3000 [37]. However, Cas9-sgRNA2 RNP did not enough induce indel mutation at the locus of sgRNA2 in *CaMLO2*. Although in silico designed guide RNAs are available throughout a reference genome, the efficacy of the designed guide RNAs needs to be validated in the aimed gene of a target genome. Due to unknown properties such as chromatin structure or epigenetic modification, of the sequence context of a target gene, we frequently observed the differential gene-editing efficiency in previous reports [18, 40, 41]. Therefore, a stable screening tool of active CRISPR tool is essential for successful crop editing.

CRISPR/Cas9-RNP in Dempsey leaf protoplasts revealed that the designed Cas9-sgRNA1 complex was 22-fold more efficient in editing *CaMLO2* compared with the Cas9-sgRNA2 complex. Similarly, Cas9-sgRNA1 RNP complex showed 88-fold higher indel frequency than did Cas9-sRNA2 RNP in CM334 callus protoplasts. The efficacy of the designed sgRNAs for Cas9 to edit *CaMLO2* in Dempsey leaf protoplasts was similar to that in CM334 callus protoplasts, although the two peppers were different cultivars. In addition, the indel patterns induced by Cas9-sgRNA1 complexes were similar to the main deletions in both Dempsey and CM334. Undeniably, it is too early to propose a general rule for the efficacy and patterns of guide RNA in pepper genome editing. However, this result raises an interesting question regarding whether a guide RNA designed for a conserved target locus has wide-ranging efficacy among pepper cultivars. This possibility can be systemically investigated at the whole genome scale.

Previously, we reported that another CRISPR-RNP tool, the LbCpf1-crRNA complex, successfully induced indels at two targeted loci of *FAD2-1A* and *FAD2-1B* in soybean [18]. Here, we also tested the activity of CRISPR/LbCpf1-RNP in pepper gene editing. We demonstrated that the efficacy of the designed crRNA1 and cRNA2 for LbCpf1 significantly differed by more than two-fold. This result indicated that CRISPR/LbCpf1-RNP precisely and effectively edited the target gene in two peppers.

To act as control experiments for the target specific editing via CRISPR RNPs, we delivered CRISPR proteins without a guide RNA, as either Cas9-only or LbCpf1-only, into pepper protoplasts. In the presence of Cas9-only for sgRNA1 and sgRNA2, and of LbCpf1-only for crRNA2, there were no induced mutations in the target loci of the *CaMLO2* gene. However, LbCpf1-only for crRNA1 locus of *CaMLO2* exhibited marginal indel frequency as shown in Fig. 5a. The repeated nucleotide sequences with 13 bp of thymine in the 3′downstream flanking region of the target crRNA1 locus may have caused the noise in indel frequency (Fig. 5a) and patterns (Fig. S1), unlike the specific indel mutation by CRISPR-crRNA1 complexes (Fig. 5a and b). The indel by LbCpf1-only in the crRNA1 locus was most likely from the error introduced by three consecutive PCR preparations performed to conduct the Next Generation Sequencing (NGS). The LbCpf1 system requires TTTN, T-rich protospacer adjacent motif (PAM) sequence in the 5′ upstream of a guide RNA (crRNA). Several *in sillico* designed crRNAs for LbCpf1 in a target genome are located at AT-rich sites or repeated sequences due to the high chance of PAM properties. Therefore, it is critical to validate whether the aimed mutation was induced at the target locus of the designed crRNA in a target gene.

Furthermore, we revealed that the editing efficacy of active guide RNAs, such as sgRNA1 for Cas9 in Dempsey leaf protoplasts, was cumulative with respect to incubation time in pepper protoplasts. Regarding the editing efficiency for *CaMLO2* in CM334, the designed Cas9-sgRNA1-RNP is comparable to the tested LbCpf1-crRNA2-RNP. The results exhibited the highest indel frequencies of 17.6% for Cas9-RNP and 19.3% for LbCpf1-RNP among the tested Cas9 and LbCpf1 RNPs. Thus, either Cas9-sgRNA1 complex or LbCpf1-crRNA2 can be used as a practical tool to edit *CaMLO2* in CM334 pepper.

Since the first seminal publications on *Arabidopsis*, tobacco, and rice in 2013 [8–10], various edited crops were obtained using CRISPR-based tools, including staple foods such as rice [8], wheat [11], soybean [18, 42], and maize [43] as well as vegetables and fruits such as tomato [44], potato [45], watermelon [46], and cabbage [40]. To the best of our knowledge, the present report provides the first data for precise pepper editing in both hot pepper and sweet pepper cultivars. We successfully edited *CaMLO2* genes in the protoplasts of two pepper cultivars with known whole-genome sequences. The established leaf or callus protoplasts are robust systems suitable to explore settled CRISPRs-RNP as well as newly developed genome editing tools for improved pepper traits.

## Conclusions

Designed DNA-free, clustered regularly interspaced short palindromic repeats (CRISPR)/ ribonucleoproteins (RNPs) screening system is a robust and prerequisite tool for precise genome editing in hot and sweet peppers.

## Methods

### Plant material and protoplast isolation

*C. annuum* cultivars CM334 and Dempsey were provided by the Vegetable Breeding Research Center (VBRC) in Republic of Korea*.* CM334 and Dempsey were germinated and grown in soil under 16 h light and 8 h dark photoperiod at 25 ± 1 °C in a growth chamber. Pepper CM334 calluses were produced in a callus inducing media (CIM, MS media contained with B5 vitamins, 3% of sucrose, 1 mg/L 2,4-dichlorophenoxyacetic acid) from fully expanded young leaves and were maintained in CIM by regular subculturing every 3 weeks [32]. The pepper calluses from the two cultivars were digested in a cell wall digesting enzyme solution [47] for 3–4 h at 24 ± 1 °C to isolate the protoplasts. The digested pepper protoplasts were diluted with an equal volume of W5 solution (154 mM NaCl, 125 mM CaCl_2_, 5 mM KCl, 5 mM glucose, 1.5 mM Mes-KOH, pH 5.6) to remove the cell wall digesting enzyme solution. The pepper protoplasts were gently collected by centrifugation at 58 *g* for 5 min, and then rinsed with W5 solution two times. The purely isolated pepper protoplasts were counted using a hemocytometer. Approximately 5 × 10^4^ isolated protoplasts were used for PEG-mediated CRISPR-RNPs delivery, as described previously [18] with slight modifications. Briefly, Cas9 or LbCpf1 proteins were premixed with an in silico designed guide RNA as a 1:6 M ratio for 1 h at room temperature. The preassembled RNP mixtures were carefully suspended with the counted protoplasts in 300 μl of MMG solution (400 mM mannitol, 15 mM MgCl_2_, 5 mM MES; pH 5.6). Subsequently, an equal volume of freshly prepared PEG solution (200 mM mannitol, 100 mM CaCl_2_, 40% PEG 4000) was added. The PEG-mixed pepper protoplasts were washed three times with an equal volume of serial dilutions using the W5 solution. They were then harvested by centrifugation at 58 g for 5 min and subsequently incubated in W5 solution. After incubation for the indicated time (24 or 48 h), CRISPR-RNPs-transfected protoplasts were prepared for genomic DNA extraction and finally analyzed for target gene editing.

### Design and preparation of guide RNA

*CaMLO2* genomic regions from the two cultivars were sequenced by Sanger sequencing to confirm the nucleotide sequences and design the guide RNAs. We used Cas-Designer of RGEN tools (http://rgenome.net) to devise specific candidate sgRNAs for Cas9 and crRNAs for LbCpf1. We obtained two sgRNAs (sgRNA1: 5′-ACATCTTCATCTGCCTTACA-3′, sgRNA2: 5′-TGATGACCCTTGTTTACAAA-3′) in the third exon for Cas9 and two crRNAs (crRNA1: 5′-TTGAACAAATTATGCATCACCTT-3′, crRNA2: 5′-GGGACACATAAGTTAGAAACTGG-3′) in the first and third exons for LbCpf1. The guide RNAs were synthesized by in vitro transcription using T7 RNA polymerase (New England Biolabs, MA, USA) as described previously [18, 24]. Primers used for in vitro transcription are listed in Table 2.

**Table 2:** Primers used in guide-RNA synthesis

Primers	Sequence (bold: guide-RNA sequences)
sgRNA1-F	GAAATTAATACGACTCACTATAG**CATCTTCATCTGCCTTACA**GTTTTAGAGCTAGAAATAGCAAG
sgRNA2-F	GAAATTAATACGACTCACTATAG**GATGACCCTTGTTTACAAA**GTTTTAGAGCTAGAAATAGCAAG
sgRNA-R	AAAAAAGCACCGACTCGGTGCCACTTTTTCAAGTTGATAACGGACTAGCCTTATTTTAACTTGCTATTTCTAGCTCTAAAAC
crRNA1-F	**AAGGTGATGCATAATTTGTTCAA**ATCTACACTTAGTAGAAATT
crRNA2-F	**CCAGTTTCTAACTTATGTGTCCC**ATCTACACTTAGTAGAAATT
crRNA-R	GAAATTAATACGACTCACTATAGGGAATTTCTACTAAGTGTAGAT

### In vitro cleavage assays using CRISPR tools

Pepper genomic DNA was prepared with a Plant SV Mini kit (GeneAll, Seoul, South Korea) and used as a template to amplify the target DNA regions of *CaMLO2*. The activity of the designed guide RNAs was validated by an in vitro cleavage assay [18]. Briefly, 240 ng of target DNA amplicons were digested with 2 μg of purified Cas9 or LbCpf1 and 1.5 μg of guide RNA in 2 μL of 10X NEB 3.1 buffer (NEB) for 1.5 h at 37 °C and subsequently incubated with RNase A for 1 h at 37 °C. The digested target DNA was purified using an MG PCR purification SV kit (MGmed, Seoul, South Korea) and analyzed by agarose gel electrophoresis.

### Targeted deep sequencing

The indel frequency and patterns were analyzed by targeted deep sequencing [18]. The prepared pepper genomic DNA was amplified with specific primers (listed in Table 3) for each guide RNA. The target amplicons were attached with multiplexing indices and specific sequencing adaptors by consecutive PCRs and subjected to high-throughput sequencing using Illumina Miseq (V2, 300 cycle) (Macrogen, Seoul, South Korea). The raw data of paired-end Miseq were analyzed using a Cas-Analyzer (http://www.rgenome.net/cas-analyzer/#!) from the RGEN tools [48].

**Table 3:** Primers used in targeted deep sequencing

Primers	Sequence
CaMLO2 F (Fig. 2a)	ATGGCTAAAGAACGGTCGAT
CaMLO2 R (Fig. 2a)	ATGGAGCTGGTGTATTGCAT
Primary F for sgRNA1, sgRNA2, crRNA2	TGGGATTCATATCATTGTTGTTG
Primary R for sgRNA1, sgRNA2, crRNA2	CCGAATGTGTCTCAGCCTTT
Primary F for crRNA1	ATGGCTAAAGAACGGTCGAT
Primary R for crRNA1	GGCACTAAGGTTGGCTACTT
Secondary F for sgRNA1, sgRNA2, crRNA2	ACACTCTTTCCCTACACGACGCTCTTCCGATCTTGGGATTCATATCATTGTTGTTG
Secondary R for sgRNA1, sgRNA2, crRNA2	ACTGGAGTTCAGACGTGTGCTCTTCCGATCTCCGAATGTGTCTCAGCCTTT
Secondary F for crRNA1	ACACTCTTTCCCTACACGACGCTCTTCCGATCTATGGCTAAAGAACGGTCGAT
Secondary R for crRNA1	GACTGGAGTTCAGACGTGTGCTCTTCCGATCTGGCACTAAGGTTGGCTACTT

The target regions of CaMLO2 edited by complexes of Cas9-sgRNA1, Cas9-sgRNA2, LbCpf1-crRNA1, or LbCpf1-crRNA2 were amplified by three consecutive PCR runs. First, the genomic region of CaMLO2 was amplified with a primer pair of CaMLO2 F and CaMLO2 R. The first PCR amplicons were used for primary PCR with primer pairs of primary F and R. The second PCR amplicons were subsequently applied by adding Illumina adaptor with primer pairs of secondary F and R. The final amplicons were subjected to bar-coding for Next Generation Sequence (NGS) analysis using Illumina Miseq

### Statistical analysis

The data are presented as the means of at least three biological replicates with standard deviation. The significant difference (*, *P* < 0.01) was assessed by one-way ANOVA with post-hoc Tukey HSD.

## Supplementary information

**Additional file 1 Fig. S1** Sequence information of *CaMLO2* crRNA1 locus in LbCpf1-only applied CM334 protoplasts. ID, ranking number of sequenced reads by NGS; Yellow, crRNA1 target sequence; Upper sequences within Sequence box, reference genomic sequence; Bottom sequence within Sequence box, sequenced reads using NGS; Misaligned – or T, evaluated as deletion or insertion. Note that LbCpf1-crRNA1 complexes that induced indel mutations are located within the yellow marked locus.

## Data Availability

The datasets used and/ or analyzed during the current study are available from the corresponding author upon reasonable request. The pepper cultivars should be requested from the Vegetable Breeding Research Center (VBRC).

## Abbreviations

Cas: CRISPR-associated protein
CRISPR: Clustered regularly interspaced short palindromic repeats
crRNA: CRISPR RNA
MLO: Mildew resistance locus O
PEG: Polyethylene glycol
RNP: Ribonucleoprotein
sgRNA: Single guide RNA
