# Supplementary figures and images for: A stable DNA-free screening system for CRISPR/RNPs-mediated gene editing in hot and sweet cultivars of Capsicum annuum
Source: BMC Plant Biol. 2020 Oct 1;20:449. doi: 10.1186/s12870-020-02665-0 (PMC7528386; doi:10.1186/s12870-020-02665-0)

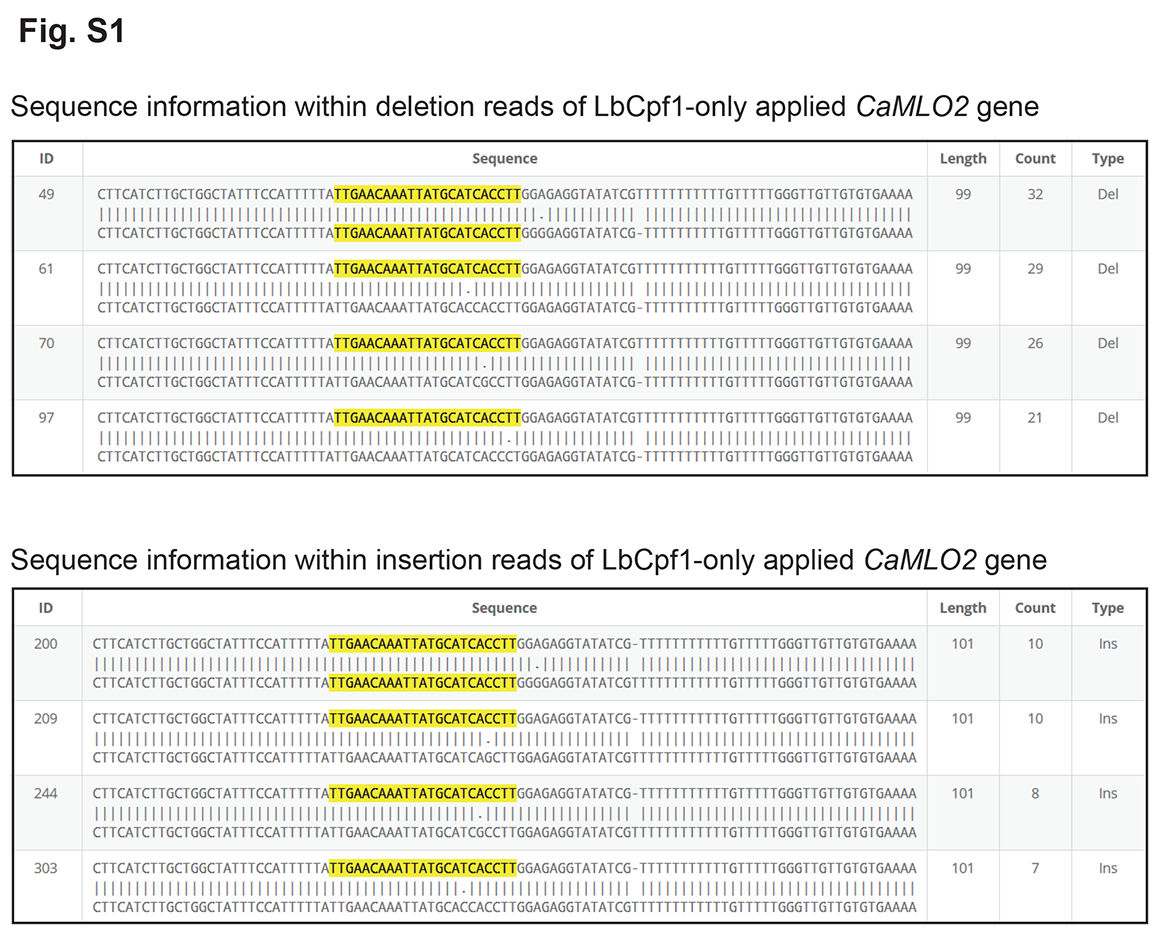

Supplement: Supplementary file 1 — Additional file 1 Fig. S1 Sequence information of CaMLO2 crRNA1 locus in LbCpf1-only applied CM334 protoplasts. ID, ranking number of sequenced reads by NGS; Yellow, crRNA1 target sequence; Upper sequences within Sequence box, reference genomic sequence; Bottom sequence within Sequence box, sequenced reads using NGS; Misaligned – or T, evaluated as deletion or insertion. Note that LbCpf1-crRNA1 complexes that induced indel mutations are located within the yellow marked locus. [file 12870_2020_2665_MOESM1_ESM.tif]
